# Factors affecting the distribution and abundance of autumn vagrant New World warblers in northwestern California and southern Oregon

**DOI:** 10.7717/peerj.5881

**Published:** 2018-12-21

**Authors:** C. John Ralph, Jared D. Wolfe

**Affiliations:** 1 Pacific Southwest Research Station, USDA Forest Service, Arcata, CA, USA; 2 Klamath Bird Observatory, Ashland, OR, USA; 3 School of Forest Resources and Environmental Sciences, Michigan Technological University, Houghton, MI, USA

**Keywords:** Migration, Age ratio, Oregon, Vagrant birds, Parulidae, Warbler, California

## Abstract

Birds found outside their typical range, or vagrants, have fascinated naturalists for decades. Despite broad interest in vagrancy, few attempts have been made to statistically examine the explanatory variables potentially responsible for the phenomenon. In this study, we used multiple linear regression to model the occurrence of 28 rare warbler species (family Parulidae) in autumn in northern California and southern Oregon as a function of migration distance, continental population size, distance, and bearing to both closest breeding population and breeding population center. In addition to our predictive model, we used capture data from the California coast to 300 km inland to examine relationships between the presence of vagrant warblers, regional warbler species richness and age class distribution. Our study yielded three important results: (1) vagrancy is strongly correlated with larger North American population size; (2) vagrants are more common at some coastal sites; and (3) where young birds are over-represented, vagrants tend to occur—such as on the coast and at far inland sites. Of the many explanations of rare and vagrant individuals, we feel that the most likely is that these birds represent the ends of the distributions of a normal curve of migration direction, bringing some few migrants to locations out of their normal migratory range as vagrants. We also examine the underrepresented species that, according to our model, are overdue for being recorded in our study area.

## Introduction

Bird observers have been long fascinated by the appearance of rare or out of range birds, and the opportunity to speculate on the causes of such vagrants.

“The erratic wanderings of migratory birds, resulting in their appearance in countries far removed from their accustomed haunts, and off the routes followed to reach them, are in many cases to be attributed to their failure, from some cause or other, to inherit unimpaired this all-important faculty of unconscious orientation. The incentive to migrate, it must be admitted, is strong within them, or they would never occur in places so remote from the domains of their respective species.”

WILLIAM EAGLE CLARKE (1912)

Coastal areas, such as northwestern California, are noted for their frequency of rare birds. The conspicuous nature of coastal vagrancy led Grinnell (1922) to predict that all species in North America would eventually turn up in California. We are, in investigating the significance and explorations of the phenomenon of vagrancy, tempted to hypothesize that the exceptions in normal migration routes that vagrants exemplify could illuminate rules that most migrants follow. For example, the regular occurrence of reverse bird migration can result in vagrancy and tends to occur among young birds during inclement weather (Nilsson & Sjöberg, 2016); these findings suggest that experience, weather, and innate directional tendencies influence broader patterns of migration. Because vagrancy generates excitement among bird observers, many prominent naturalists have speculated on the mechanisms responsible for the occurrence of rare birds: weather, geography, migration overshoots, deviant directional tendencies, mirror-image migration, and reversed direction migration (Rabøl, 1976; Burger, Williams & Sinclair, 1980; McLaren, 1981; DeSante, 1983a; Montalti, Orgeira & Di Martino, 1999; Thorup, 2004; Newton, 2008). Despite interest in publishing theories regarding the causes of vagrancy, relatively few attempts (Hampton, 1997) have been made to statistically examine the influence of multiple explanatory factors on the prevalence of vagrancy at the landscape scale.

Similar to vagrancy, a disproportionately high number of young birds (<6-months-old) are regularly observed during fall migration along California’s northwest coast. The preponderance of young birds found in coastal regions was termed the “coastal effect” by Ralph (1978, 1981). It was suggested that the coastal effect is a manifestation of misoriented young nocturnal migrants being forced to return to land at sunrise after traveling above the ocean, resulting in more young birds near coastal areas (Ralph, 1978). Similarities between coastal vagrancy and the coastal effect are striking, where young and vagrant birds appear to be relatively more common near the coast, and may simply reflect the misorientation of both young of many species and vagrant birds. More specifically, if young birds are more prone to becoming lost (similar to a lost vagrant bird), then there will be a correlation between the abundance of young and vagrant birds at geographic boundaries, such as oceans and deserts, that prevent passage across the landscape. Furthermore, if vagrant species are more abundant along coastlines, then coastal communities should be more species rich when compared to their inland counterparts. In this study, we conducted three analyses to explore factors influencing the presence and richness of vagrant and young birds across northern California and southern Oregon.

First, we used vagrant warbler records from northern California to examine the relative importance of six explanatory factors in illuminating the likely causes of vagrancy in New World warblers (Parulidae). Based on these findings we provided a method of predicting which warbler species have likely been missed by observers. Second, we used capture data from northern California and southern Oregon to determine if vagrancy asymmetrically influenced warbler species richness across the landscape. Third, we used capture data to test the prediction that young and vagrant warblers probably represent lost birds subject to the vagaries of geography, and their abundances are, therefore, statistically correlated across the landscape.

## Methods

**Figure 1:**
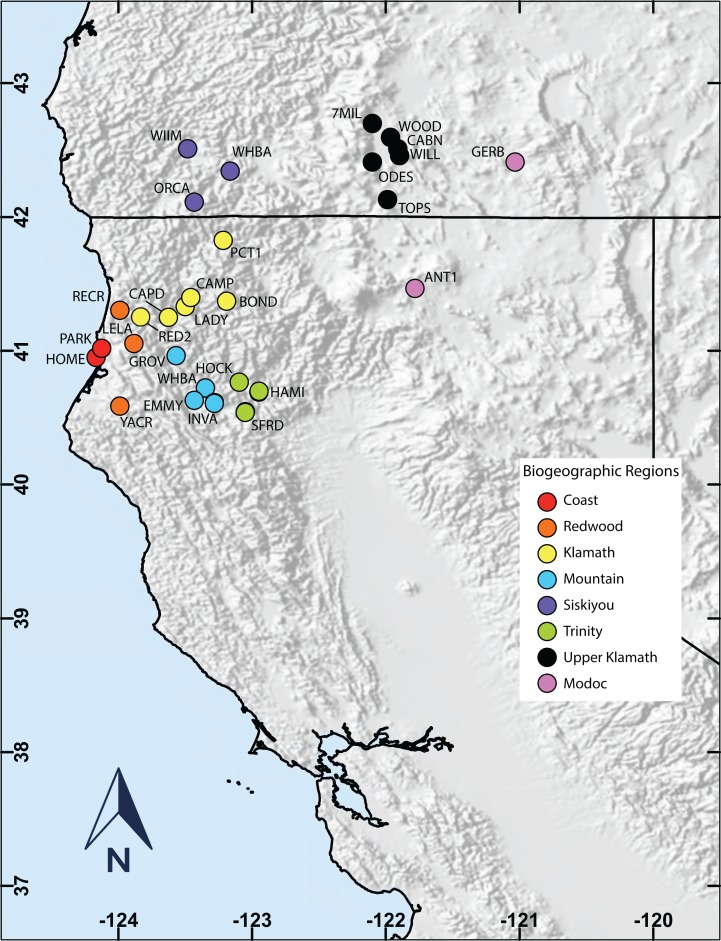
Map of the study area. Location of capture stations across the study area, and further divided into biogeographical regions based on similarities of distance to the coast, altitude, and habitat.

Vagrancy is a site-specific phenomenon, as one area’s common bird is another’s rare vagrant, or out of range species, so we limited our study to two overlapping study areas. The first study area is delineated by the 28 bird banding stations used in this study (from our network of 239 banding stations known as the Klamath Bird Monitoring Network; Alexander & Ralph, 2004) in northern California and southern Oregon (Fig. 1). The second study area is based on the meticulous compilation of all available records from all observers in northwestern California by Harris (2006) that includes the western half of Siskiyou County, all of Del Norte, Humboldt, and Trinity counties, and the northern half of Mendocino County. From this, we retrieved fall sightings (September–November) of all vagrant warbler species (Table 1) documented from 1970 to 2006.

**Table 1:** Variables used in predictive vagrant warbler models.

Species	Migratory distance	North American population	Fall records	Distance to closest breeding range (km)	Bearing to closest	Distance to center of population (km)	Bearing to center
American Redstart (AMRE; *Setophaga ruticilla*)	31.38	25,000,000	343	400	41	1,800	63
Bay-breasted Warbler (BBWA; *Setophaga castanea*)	39.22	3,000,000	25	1,000	7	1,700	56
Black-and-white Warbler (BAWW; *Mniotilta varia*)	34.09	14,000,000	247	800	32	2,000	70
Blackburnian Warbler (BLBW; *Setophaga fusca*)	46.08	5,900,000	27	1,000	31	2,200	68
Blackpoll Warbler (BLPW; *Setophaga striata*)	53.28	20,000,000	208	800	358	1,700	37
Black-throated Blue Warbler (BTBW; *Setophaga caerulescens*)	23.83	2,000,000	51	1,700	62	2,400	66
Black-throated Green Warbler (BTNW; *Setophaga virens*)	30.34	10,000,000	14	900	19	2,000	70
Blue-winged Warbler (BWWA; *Vermivora cyanoptera*)	34.09	390,000	3	1,500	84	2,200	80
Canada Warbler (CAWA; *Cardellina canadensis*)	47.88	1,400,000	14	900	20	2,000	66
Cape May Warbler (CMWA; *Setophaga tigrina*)	36.39	3,000,000	18	900	12	1,700	55
Cerulean Warbler (CEWA; *Setophaga cerulea*)	41.44	560,000	1	1,400	82	2,200	81
Chestnut-sided Warbler (CSWA; *Setophaga pensylvanica*)	29.51	9,400,000	134	1,000	25	2,200	65
Connecticut Warbler (COWA; *Oporornis agilis*)	53.21	1,200,000	3	1,000	25	1,500	48
Golden-cheeked Warbler (GCWA; *Setophaga chrysoparia*)	16.01	21,000	0	1,600	109	1,600	72
Golden-winged Warbler (GWWA; *Vermivora chrysoptera*)	33.32	210,000	3	1,300	47	2,200	72
Grace’s Warbler (GRWA; *Setophaga graciae*)	11.84	1,000,000	0	600	127	1,800	114
Hooded Warbler (HOWA; *Setophaga citrina*)	18.80	4,000,000	27	1,500	82	2,200	85
Kentucky Warbler (KEWA; *Geothlypis formosa*)	19.87	1,100,000	4	1,500	92	2,000	87
Kirtland’s Warbler (KIWA; *Setophaga kirtlandii*)	20.62	2,100	0	2,000	72	2,000	132
Louisiana Waterthrush (LOWA; *Parkesia motacilla*)	20.97	260,000	0	1,500	94	2,100	110
Lucy’s Warbler (LUWA; *Oreothlypis luciae*)	11.57	900,000	5	600	128	1,000	126
Magnolia Warbler (MAWA; *Setophaga magnolia*)	33.92	30,000,000	75	700	11	1,800	57
Mourning Warbler (MOWA; *Geothlypis philadelphia*)	43.44	7,000,000	2	900	20	2,000	66
Northern Parula (NOPA; *Setophaga americana*)	19.88	7,300,000	89	1,000	90	1,800	84
Northern Waterthrush (NOWA; *Parkesia noveboracensis*)	40.57	13,000,000	101	400	58	1,500	39
Olive Warbler (OLWA; *Peucedramus taeniatus*)	0.00	17,000	0	900	119	1,800	129
Ovenbird (OVEN; *Seiurus aurocapilla*)	26.05	24,000,000	34	800	56	1,900	74
Painted Redstart (PARE; *Myioborus pictus*)	8.82	40,000	0	700	124	1,800	94
Pine Warbler (PIWA; *Setophaga pinus*)	12.13	11,000,000	0	1,500	56	2,200	78
Prairie Warbler (PRWA; *Setophaga discolor*)	15.69	1,400,000	53	1,500	91	2,100	86
Prothonotary Warbler (PROW; *Protonotaria citrea*)	20.39	1,800,000	25	1,500	101	2,100	89
Red-faced Warbler (RFWA; *Cardellina rubrifrons*)	0.00	110,000	0	700	124	1,100	126
Swainson’s Warbler (SWWA; *Limnothlypis swainsonii*)	12.24	84,000	0	1,700	108	2,100	84
Tennessee Warbler (TEWA; *Leiothlypis peregrina*)	42.47	60,000,000	144	700	33	1,700	51
Tropical Parula (TRPA; *Setophaga pitiayumi*)	31.38	3,000	0	1,200	131	3,800	128
Virginia’s Warbler (VIWA; *Leiothlypis virginiae*)	18.53	400,000	15	300	119	800	107
Worm-eating Warbler (WEWA; *Helmitheros vermivorum*)	19.43	700,000	10	1,500	82	2,200	86
Yellow-throated Warbler (YTWA; *Setophaga dominica*)	16.71	1,600,000	20	1,500	82	2,200	88

**Note:**

Variables used to predict autumn vagrancy of warblers (recorded fewer than 500 times) in northwestern California (from Harris, 2006).

We defined a vagrant as a species that was detected at least once, and with a maximum of a total of 500 records, over a 36 year period (Harris, 2006). We felt that this was a useful number that differentiated relatively rare migrant birds from vagrant species that are occurring outside their normal range. For example, we did not consider the Palm Warbler (see Table 1 for scientific names) a vagrant, but a regular, yet rare, migrant with some 1,100 fall records. We found that 28 warbler species could be classified as vagrants because of their rarity (Table 1).

For our predictive model, the response variable was a log transformation (to normalize the distribution) of the total number of individuals of each vagrant species detected during fall migration from Harris (2006). For example, Black-throated Blue Warbler had 51 and Blue-winged Warbler had three individuals documented during fall migration in northern California; each of these values represented a single datum. Explanatory variables were chosen based on their probability of affecting vagrant warbler occurrence in northern California and southern Oregon. Broadly, we included explanatory variables that measured three influential factors: migratory distance, population size, and direction to breeding population (Thorup, 2004; McLaren et al., 2006). Based on these three factors, we selected six explanatory variables to explain fall vagrant warbler sightings: migration distance, size of the North American breeding population (log), distance to closest population (log), bearing to closest population, distance to population center (log), and bearing to population center (Pfeifer, Stadler & Brandl, 2007). Distances were estimated by taking digital measurements from the centroid of our study area to the edge of the closest breeding range, as well as the center of the breeding population. Migration distance class was calculated by the number of degrees latitude between the breeding and wintering ranges using BirdLife range maps (http://www.birdlife.org/datazone/species). Our use of degrees latitudes likely underestimated migrant birds that move across longitudes as well as latitudes. However, given that long-distance warbler migration is characterized by changes in latitude, we felt comfortable that degrees latitude serves as an excellent index of migratory distance among our study species. We also calculated distance to closest population, bearing to closest population, distance to population center, and bearing to population center. The inclusion of distance and bearing to closest breeding population accounted for distant, yet westerly populations of breeding vagrants. Distance estimates were to the nearest 100 km, from the combined center of the four counties of our study area to the center of each species’ breeding range. North American population estimates were taken from Rich et al. (2004).

We formulated 16 competitive multiple-linear regression models (Table 2), including a null (no explanatory variables) and global model (all explanatory variables) using program R (R Core Team, 2012). Models were formulated a priori based on a combination of explanatory variables we believed most influenced vagrancy. Next, we ranked each model using Akaike information criterion values corrected for small sample sizes (AICc) where the top model was selected if it was at least two AICc values lower, and/or had fewer parameters relative to the next most competitive model (Arnold, 2010). We also included 95% confidence intervals with explanatory variable beta estimates and adjusted *R*^2^ with each model to provide additional information regarding how well each model performed and fit the data. Once the top model was selected, we used covariate data from ten other warbler species (Table 1) that had not yet been documented in the study area, but were possible candidates for future vagrant status (Tropical Parula, Olive Warbler, Painted Redstart, Pine Warbler, Grace’s Warbler, Red-faced Warbler, Swainson’s Warbler, Golden-cheeked Warbler, Louisiana Waterthrush and Kirtland’s Warbler), to predict which species were most likely to occur in northern California.

**Table 2:** Predicitive model rankings.

Model	ΔAICc	*w*_AICc_	Deviance	*k*	adj. *R*^2^
Migratory distance + North American population	0.00	0.44	25.92	3	0.56
Migratory distance + North American population + distance to closest breeding population + bearing to closest breeding population + distance to center of breeding population + bearing to center of breeding population	0.68	0.31	16.20	7	0.67
North American population	1.53	0.20	30.19	2	0.50
North American population + distance to closest breeding population + bearing to closest breeding population	5.08	0.03	27.94	4	0.50
North American population + distance to center of breeding population + bearing to center of breeding population	6.87	0.01	29.78	4	0.47
Migratory distance + distance to center of breeding population + bearing to center of breeding population	13.84	0.00	38.19	4	0.32
Distance to closest breeding population + bearing to closest breeding population + distance to center of breeding population + bearing to center of breeding population	15.04	0.00	35.47	5	0.34
Distance to closest breeding population	16.71	0.00	51.93	2	0.15
Bearing to center of breeding population	19.00	0.00	56.34	2	0.07
Distance to closest breeding population + bearing to closest breeding population	19.13	0.00	51.34	3	0.12
Null	19.72	0.00	63.25	1	n/a
Distance to center of breeding population + bearing to center of breeding population	21.26	0.00	55.40	3	0.05
Migratory distance + distance to closest breeding population + bearing to closest breeding population	21.76	0.00	50.67	4	0.10
Bearing to closest breeding population	21.95	0.00	62.61	2	0.00
Distance to center of breeding population	22.20	0.00	63.17	2	0.00
Migratory distance	22.21	0.00	63.19	2	0.00

**Note:**

Candidate models used to predict warbler vagrancy in northern California and associated differences in corrected AIC values (ΔAICc), AICc weights (*w*_AICc_), model deviance, number of parameters (*k*), and adjusted *R*^2^ values. The most competitive model was selected if it was at least two AICc values lower, and/or had fewer parameters relative to the next most competitive model (Arnold, 2010).

Next, we used capture and banding data from 28 stations across southern Oregon and northern California, representing eight geographic regions, to explore relationships between location and warbler species occurrences. Each of the stations had at least a total of 10,000 mist-net hours of banding data during fall migration, and for convenience, were subjectively grouped into regions based on empirical criteria of distance to coast, latitude, altitude, and habitat (Fig. 1; Table 3). Most nets were amongst riparian vegetation to achieve higher capture rates, irrespective if they were near oak woodland or dense coniferous forest.

**Table 3:** Capture summaries of vagrant and non-vagrant warblers.

			Non-vagrant			Vagrant		
Region	Site	Mist-net hours	*n*	%HY	%Unk	*n*	%HY	%Unk
Coast	PARK	14,748	446	0.84	0.25	5	0.8	0
Coast	HOME	51,845	2,494	0.85	0.19	34	0.88	0
**Coast total**		**66,593**	**2,940**	**0.85**	**0.22**	**39**	**0.84**	**0**
Redwood	RECR	3,809	110	0.58	0.08	0	n/a	n/a
Redwood	LELA	1,495	44	0.65	0.02	0	n/a	n/a
Redwood	YACR	2,769	70	0.68	0.06	0	n/a	n/a
**Redwood total**		**8,072**	**70**	**0.68**	**0.06**	**0**	**n/a**	**n/a**
Klamath	CAPD	9,739	453	0.87	0.09	0	n/a	n/a
Klamath	RED2	4,258	287	0.78	0.08	2	1	0
Klamath	CAMP	5,944	352	0.83	0.05	0	n/a	n/a
Klamath	LADY	6,564	553	0.77	0.07	1	1	0
Klamath	BOND	658	54	0.79	0.04	0	n/a	n/a
Klamath	PCT1	10,775	735	0.84	0.09	1	1	0
**Klamath total**		**37,939**	**2,434**	**0.85**	**0.07**	**4**	**1**	**0**
Mountain	EMMY	2,449	69	0.55	0.04	0	n/a	n/a
Mountain	INVA	7,364	1,134	0.65	0.1	0	n/a	n/a
Mountain	GROV	8,219	1,021	0.79	0.12	1	1	0
**Mountain total**		**18,031**	**2,224**	**0.66**	**0.09**	**1**	**1**	**0**
Siskiyou	ORCA	2,790	300	0.83	0.07	0	n/a	n/a
Siskiyou	WHBA	3,808	73	0.54	0.03	0	n/a	n/a
Siskiyou	WIIM	16,467	2,529	0.74	0.04	0	n/a	n/a
**Siskiyou total**		**23,065**	**2,902**	**0.7**	**0.05**	**0**	**n/a**	**n/a**
Trinity	HOCK	3,395	213	0.53	0.38	1	0	1
Trinity	SFRD	3,564	98	0.49	0.27	0	n/a	n/a
Trinity	HAMI	2,381	168	0.74	0.14	0	n/a	n/a
**Trinity total**		**9,340**	**479**	**0.58**	**0.26**	**1**	**0**	**1**
Upper Klamath	TOPS	4,307	491	0.75	0.08	0	n/a	n/a
Upper Klamath	ODES	5,605	1,073	0.81	0.1	5	0.2	0
Upper Klamath	CABN	13,803	3,958	0.83	0.13	2	1	0
Upper Klamath	7MIL	6,569	446	0.84	0.25	0	n/a	n/a
Upper Klamath	WOOD	5,130	1,229	0.83	0.11	0	n/a	n/a
Upper Klamath	WILL	5,394	1,434	0.68	0.15	2	1	0
**Upper Klamath total**		**40,809**	**8,631**	**0.79**	**0.14**	**9**	**0.73**	**0**
Modoc	ANT1	4,152	1,640	0.75	0.1	1	0	0
Modoc	GERB	4,496	431	0.74	0.12	0	n/a	n/a
**Modoc total**		**8,648**	**2,071**	**0.74**	**0.11**	**1**	**0**	**0**
**Total**		**203,851**	**21,751**	**0.73**	**0.13**	**55**	**0.6**	**0.15**

**Note:**

Number captured (*n*), percent young (%HY), percent unknown age (%Unk) of vagrant and non-vagrant warblers detected during fall migration at each capture station (site), grouped by biogeographic region (region), between 1992 and 2008. The bold face rows are totals for each of the regions.

To estimate the number of species of both all warblers and vagrant warblers, we generated Chao1 estimates of total and vagrant warbler richness, using program EstimateS (Colwell, 2005), at each banding station to examine the influence of vagrancy on warbler richness across southern Oregon and northern California. The Chao1 diversity index uses the ratio of species detected only once or twice to generate predicted estimates of species richness. The formula used for Chao1 estimates are based on Chao (1987) where *S*_observations_ refers to total number of species observed in all samples pooled and *F*_1_ and *F*_2_ refer to species detected only once or twice:

$${\hat S_{{\rm{chao}}1}} = {S_{{\rm{observations}}}} + \left({{{n-1} \over n}} \right){{{F_1}^2} \over {2{F_2}}}$$

Finally, we used the same Klamath banding dataset (described above) to test the hypothesis that the proportion of young birds and vagrant bird abundances are correlated across the landscape (DeSante, 1973; Ralph, 1981). To examine this hypothetical relationship, we performed a simple linear regression to examine the correlation between the percent of all warbler species pooled that were young, and the abundance of vagrant warblers captured per 1,000 net-hours across the eight geographic regions in southern Oregon and northern California during fall migration.

## Results

### Factors affecting the occurrence of vagrants

**Figure 2:**
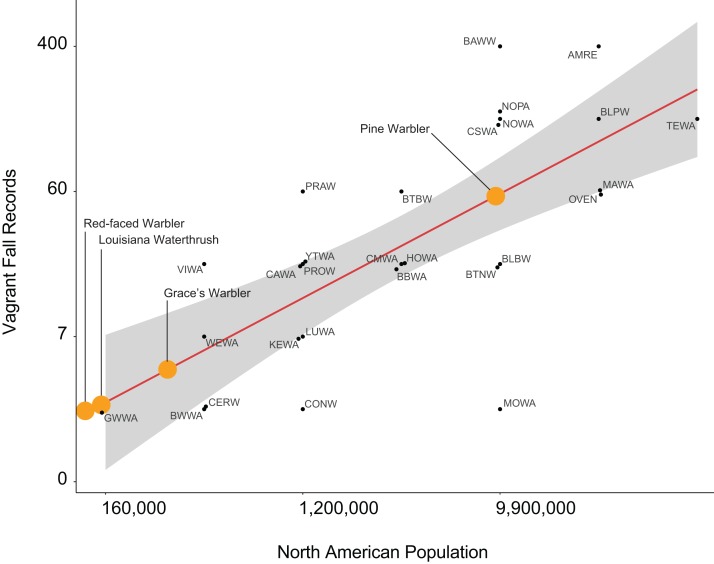
Visualization of the top model predicting vagrant warbler occurrence. Visualization of our top model demonstrating a positive correlation between North American population size and number of vagrants detected in northern California (taken from Harris, 2006). Each four-letter code represents the AOU short-hand abbreviation for each warbler species. Orange marks indicate predicted occurrence of yet unrecorded vagrant warbler species in northern California; six unrecorded vagrant warbler species have small populations and thus do not appear in this figure as they would fall below, or well below, an observation of a single individual predicted: Tropical Parula, Painted Redstart, Olive Warbler, Swainson’s Warbler, Golden-cheeked Warbler, and Kirtland’s Warbler.

Using the number of fall records of the 28-vagrant species as a response variable (Table 1), we investigated combinations of six likely explanatory variables. We selected the top model based on being within two AICc values of the lowest value, while having the fewest number of parameters (Arnold, 2010). Based on these criteria, we selected North American population size as the most competitive explanatory variable explaining vagrancy in northern California (Table 2). Specifically, we found that vagrancy was positively correlated with the North American breeding population (Fig. 2), indicating that the larger the species’ breeding population, the more likely it is to occur in northern California as a vagrant (β = 0.719; SE = 0.135; CI = [0.45–0.98]). Based on our model selection criteria (Arnold, 2010), our second most competitive model yielded the lowest AICc value and contained migration distance as an additional explanatory variable (β = −0.035; SE = 0.017; CI = [−0.001 to −0.069]). Together, the two most competitive models encompassed 64% of the AICc weight, and each explained 56% of the variance. The next most competitive model had the second lowest AICc value and included two-additional parameters where beta confidence intervals did not overlap zero, suggesting species with nearer breeding populations (β = −2.154; SE = 0.84; CI = [−0.504 to −3.804]) coupled with more direct bearings to breeding range centers (β = −0.036; SE = −0.018; CI = [−0.0003 to −0.072]) exhibited more vagrancy. This latter model contained two other additional explanatory variables where beta estimate confidence intervals overlapped zero and were therefore deemed not significant: distance to center of breeding population (β = 2.66; SE = 1.50; CI = [−0.27–5.60]), and bearing to center of breeding population (β = −0.036; SE = 0.018; CI = [−0.076–0.003]).

### Species recorded less or more often than expected

We detected a strong linear relationship between the number of fall records for the 28 species and the size of their North American breeding population, with most falling near the regression line (Fig. 2). The possibly instructive exceptions include those underrepresented, seen much less often than the population size would predict, with their values below the regression line (i.e, the largest negative residuals), including Connecticut Warbler, Cerulean Warbler, and Mourning Warbler. Conversely, “overrepresented” species tending to be recorded more often than would be predicted (the largest positive residuals) included Prairie Warbler, Virginia’s Warbler, Black-and-White Warbler, and Black-throated Blue Warbler.

We included the predicted number of records of 10 species of warblers that have never been recorded in northern California to identify what species, according to our model, should have been detected (Fig. 2). Painted Redstart, Swainson’s Warbler, Red-faced Warbler, and Louisiana Waterthrush would have been predicted to be detected fewer than three times, but Grace’s Warbler had ∼7 predicted records and Pine Warbler possibly more than 50 records. The other four species with no records had populations of less than 40,000 and would have a low probability of occurring in northwest California.

### Influence of vagrancy on warbler species richness

**Figure 3:**
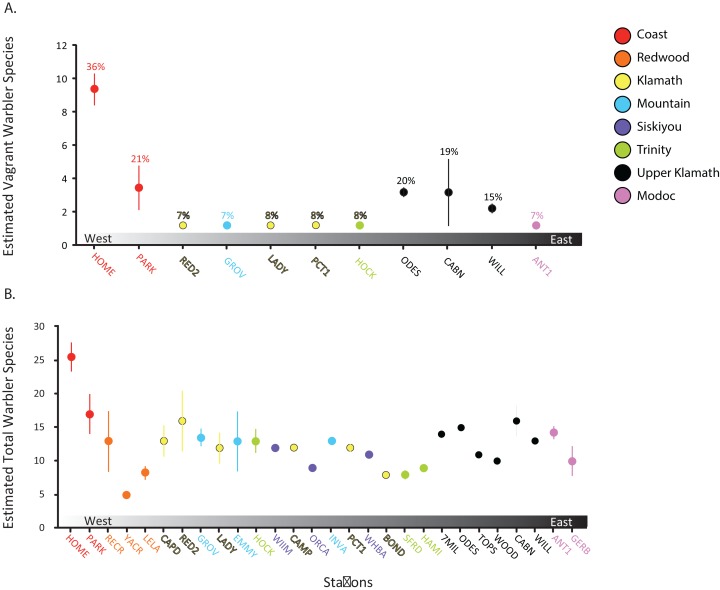
Diversity estimates of warblers at banding stations. Estimated number of vagrant warbler species (A) at the 11 stations with at least one species of vagrant recorded; percent values indicate the proportion of warbler species richness represented by vagrants. Estimated total warbler species (B) at all 28 bird capture stations, with standard error bars, and grouped (colored) by the eight biogeographic regions in southern Oregon and northern California between 1992 and 2008.

When comparing the number of warbler species at the 11 stations that had at least one vagrant species occurring (Fig. 3), we found that vagrant warblers were most common at the HOME station (in the Coastal Region) where vagrant warblers accounted for 9 of the 26 estimated species (36%). This was our highest ratio of vagrant to total species; this finding indicates that vagrancy strongly influenced total warbler richness at our most species-rich site. Conversely, vagrants at inland stations generally accounted for a smaller proportion of total warbler species richness, with 17 stations having none or relatively few vagrants. These species-poor stations could be located just inland from the coast, such as 7% at the RED2 station in the Redwood Region, only 4.5 km from the coast. At the much farther inland sites, however, the estimated proportion of vagrants rose again sharply to 19% at CABN and 20% at ODES on the western shore of Upper Klamath Lake in the Upper Klamath Region (Fig. 3).

### Associations between young birds of all species and vagrants

**Figure 4:**
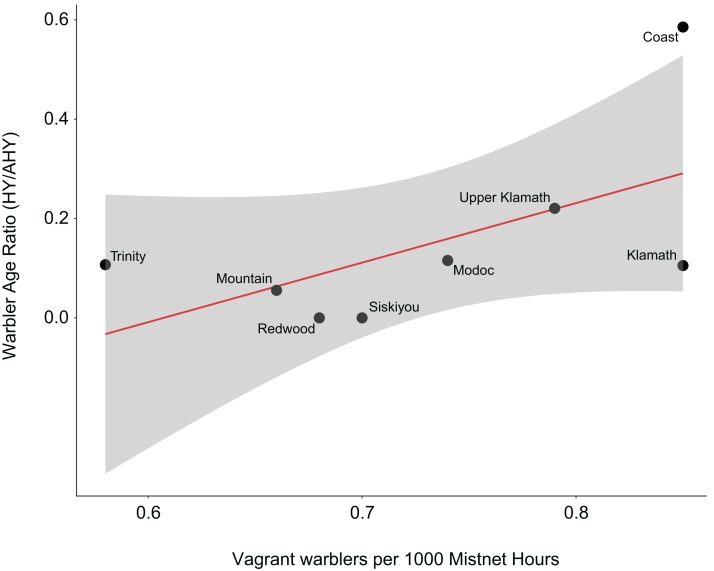
Relationship between vagrant and young warblers. Visualization of our linear regression examining the relationship between the number of vagrant warblers and proportion of non-vagrant warbler age ratios (hatching year [HY]/after-hatching year [AHY]). Data were collected from 28 bird capture stations, across eight biogeographic regions, operated in southern Oregon and northern California between 1992 and 2008.

Previous evidence suggests that banding stations along coasts have a high proportion of young birds (the “coastal effect” of Ralph, 1971, 1978, 1981). To examine if both young birds in general, and vagrant abundances, are similarly over-represented in certain regions across the study area, we employed linear regression using percent of young (all non-vagrant warblers pooled) as the response and vagrant warbler abundance captured across the regions as the explanatory variables. Our analysis found a moderately-significant relationship between the percent of young birds and the abundance of vagrant warblers captured across the eight geographic regions. Specifically, while beta estimate confidence intervals slightly overlapped zero (β = 0.299; SE = 0.163; CI = [0.02–0.62]), associated adjusted *R*^2^ values were high, where vagrant records explained 25% of the variance in non-vagrant age ratios. Thus, areas with higher proportions of young warblers tended to have more vagrant warblers. As expected, the region with both the highest proportion of young and the highest proportion of vagrant warblers was the coastal region (Fig. 4; Table 3).

## Discussion

Our analysis revealed three major insights: (1) vagrancy is largely driven by large population size—species with more individuals increased the likelihood of being detected outside a species’ normal range. This is a logical confirmation and extension of other studies (Stake, 2012); (2) vagrancy drives warbler richness in some coastal sites and does not affect warbler richness at other, more inland sites; and (3) stations where young warblers account for a higher proportion of total captures, vagrant warblers also tend to occur—such as on the coast. Our findings suggest that young and vagrant birds have something in common: they both may be more prone to navigational mistakes, and therefore more often occur on the coast, an inherently dangerous place for these nocturnal migrants.

In an early analysis of California warblers, DeBenedictis (1971) also presented some subjective evidence that continental population size accounted for some variation in abundance of vagrants in California. He also suggested involvement of the angle of deviation from normal migration routes needed to reach California. By contrast, DeSante (1983b) suggested that on the Farallon Islands, some 30 km offshore, the abundance of vagrant warblers might be related to their respective commonness in North America. Another analysis by Hampton (1997) examining patterns of vagrancy relied on a linear model to explore factors that may have influenced the occurrence of 84 rare species throughout coastal and central northern California. Of the six explanatory variables included in a linear model, he found support for the following variables in explaining the occurrence of vagrants: westernmost longitude of a species’ breeding range, taxonomic status, distance to the nearest edge of the breeding range, and easterly migratory route. Interestingly, unlike our findings, Hampton found little support for population size. Differences in Hampton’s (1997) and our findings may reflect statistical methodologies, that is, we ranked a series of competitive models using AICc, while Hampton explored a single model. Our study area and taxonomic unit, wood warblers in northern California, was also much smaller relative to Hampton’s that included all vagrant landbirds across much of the state.

Interestingly, we found four warbler species with no records in northern California (Harris, 2006) that, according to our model, should have been recorded at least once. Two species would be predicted to have a single individual Louisiana Waterthrush and Red-faced Warbler; but two others would be much more abundant: Grace’s Warbler with seven records and the Pine Warbler with 45 records predicted. However, the facultative migratory behavior of the Pine Warbler—meaning some populations may not migrate—could be partly responsible for the overestimation of their potential occurrence in our study area (F. Moore, 2018, personal communication). Assuming that our predictive model is robust, both Grace’s Warbler and Pine Warbler might not have occurred in our study area because they are more accurate in their navigation, or they could have been missed by observers because of identification problems, as both species are relatively cryptic. Pine Warbler resembles the somewhat more common Blackpoll Warbler, and other species, such as the Orange-crowned Warbler (*Oreothlypis celata*). Interestingly, Pine Warbler has been detected south of the study area along the central coast of California, thereby suggesting the species has likely occurred in our study area (see *ebird* citizen science records: https://ebird.org/map/pinwar). Grace’s Warbler is a southwestern species, and may be confused with immature Townsend’s Warbler (*Setophaga townsendi*), a common species in the study area. Several other species appear to be relatively under-detected, and below the trend line. These are generally either cryptic or hard to detect (e.g., Mourning Warbler, underrepresented by almost an order of magnitude, and Connecticut Warbler both of which are likely confused with the common migrant MacGillivray’s Warbler (*Geothlypis tolmiei*). In addition to appearing similar to MacGillivray’s Warbler, Connecticut Warbler prefers thick vegetation near the ground making them inherently difficult to find (Pitocchelli et al., 2012). According to our model, other underrepresented vagrants include Ovenbird and Cerulean Warbler, both of which may be challenging to detect because Ovenbirds prefer forested understories and the Cerulean Warbler frequents forest canopies—an arduous stratum to look for birds in the often-towering trees of the north coast. By contrast, overrepresented species, seen relatively more often than their population size would indicate, are typically conspicuous, or easily identified species, such as Black-and-white Warbler, Prairie Warbler, Chestnut-sided Warbler, American Redstart, and Black-throated Blue Warbler. It is also possible that, in addition to being conspicuous, these overrepresented species may be more prone to misorientation or have produced more young during the years of this study, relative to other species, thereby increasing the probability of young vagrants being detected on the coast.

A high proportion of young birds on the Pacific coast, the “coastal effect” (Ralph, 1978, 1981) is suggested to be primarily due to misorientation of the relatively naïve young individuals at the edge of their migratory flyway. Several hundred kilometers farther inland is another periphery of many migrant species’ routes, the eastern portion of our study area, where the habitat is less salubrious, and on the eastern edge of a major habitat type, the coniferous Cascade Mountains, and on the western edge of the relatively inhospitable Great Basin. In this portion of our study area, we found an increase in the number of vagrants, but did not find a concomitant high proportion of young. The increase of vagrants here along the western shore of Upper Klamath Lake may be related to this shoreline, acting as a “coast” analogous to the shore of the Pacific Ocean. In addition, it is quite possible that young birds on the coast may be due to one phenomenon, and the occurrence of vagrants might be associated with a different phenomenon. For example, Austin (1971) found that migrants which breed east of the Rockies typically occur approximately three weeks later in migration than migration dates in the east. A high percentage of these lost vagrants were immature. He suggested that they were transported westward, by airflows from east across the southwestern states, that could have played a role, as well as misorientation. However, DeSante (1973) clearly found that vagrant wood warblers in California occurred on time, relative to their average timing on their normal migration route.

While today’s vagrant might be tomorrow’s model citizen, destined to become a colonizer and perhaps an established resident, as Grinnell (1922) asserted, most vagrants might be viewed as “failed colonization attempts”. Newton (2008: 267–299) summarized quite well the various explanations of the causes of vagrancy put forward over the past century or so. They include: normal dispersal over long distances, population growth or expansion, drift by winds, migration overshoots, deviant directional tendencies (right time but wrong direction), mirror-image migration, and reversed direction migration. While all explanations probably play a role and explain the occurrence of some vagrant individuals, we address the latter three explanations as they likely involve the vast majority of landbirds. The mirror-image misorientation theory, originally developed by DeSante (1973), and described by Diamond (1982), proposed that vagrants are misoriented by confusion of right and left in relating an inherited migration direction to a compass reference direction. Mirror-image misorientation theory accounts for observations made by DeSante (1983a) that in certain situations large-angle misorientations seem more frequent than small or intermediate deviations from the normal migration course (Alerstam, 1990). Misorientation by the wind has long been suggested as a cause of accidentals (Austin, 1971), but Thorup et al. (2012) found differently, as the authors used radio telemetry to track individual migratory flights of several species of songbirds from the Faroe Islands, approximately halfway between Norway and Iceland, far west of their normal migration route. Birds with expected easterly and south-easterly migration direction departed westward out over the Atlantic Ocean, indicating that these birds are actively flying in the “wrong” direction and that their occurrence is not caused by wind drift. However, on Attu Island, in the Aleutian Islands off Alaska, Hameed et al. (2009) found statistical evidence that the occurrence of spring Asian vagrants on this North Pacific island were correlated with storm winds from the west.

Perhaps none of our proposed explanatory variables captured the essence of either simple misorientation or mirror-image misorientation in determining the abundance of vagrants, because they do not include information on the normal fall migration routes of the various species. For example, American redstart and Black-and-white Warbler may be more abundant than predicted from source populations because they commonly winter in southern Baja California, much farther west than other species with similar breeding ranges, and thus require much smaller degrees of simple misorientation from their normal fall migration route to reach northern California. Similarly, Prairie, Black-throated Blue and Blackpoll Warblers may be more abundant than predicted due to their easterly fall migration routes which facilitates mirror-image vagrants in California.

We would advance that perhaps a more parsimonious explanation of the phenomenon, and certainly part of some of the mechanisms listed above, is that these vagrants represent the ends of the distributions of a normal curve of the migration direction of the species, thus bringing some few migrants to unaccustomed locations. As to the other explanations that have been advanced, as Newton (2008: 299) notes “possible bias in observer coverage throws doubt on some apparent examples of mirror-image and reversed-direction migration, and neither mechanism can be considered as proven or disproven”. Our explanation of misorientation of young along the coast as being the result of deviant directional tendencies remains well-demonstrated (Ralph, 1978). Hence, it remains quite likely that the probability of an individual migrant suffering from deviant directional tendencies increases with population size, leading to our documented correlation between the abundance of vagrant warblers and total population size.

## Additional Information and Declarations

### Data Availability

The following information was supplied regarding data availability: Datasets used (Humboldt Bay Bird Observatory and Klamath Bird Observatory) were downloaded from the Avian Knowledge Northwest site (http://www.avianknowledgenorthwest.net/).
